# Complex Structural *PPT1* Variant Associated with Non-syndromic Canine Retinal Degeneration

**DOI:** 10.1534/g3.118.200859

**Published:** 2018-12-12

**Authors:** Leonardo Murgiano, Doreen Becker, Dina Torjman, Jessica K. Niggel, Ausra Milano, Cheryl Cullen, Rui Feng, Fan Wang, Vidhya Jagannathan, Sue Pearce-Kelling, Martin L. Katz, Tosso Leeb, Gustavo D. Aguirre

**Affiliations:** * Department of Clinical Sciences & Advanced Medicine, School of Veterinary Medicine, University of Pennsylvania, Philadelphia, PA; † Institute of Animal Breeding and Husbandry, University of Kiel, Germany; ‡ OptiGen, LLC, Cornell Business & Technology Park, Ithaca, NY; § CullenWebb Animal Eye Specialists, Riverview, N.B Canada; ** Department of Biostatistics, Epidemiology and Informatics, University of Pennsylvania Perelman School of Medicine, Philadelphia, PA; †† Department of Molecular Cardiology, Cleveland Clinic Lerner Research Institute, Cleveland, OH; ‡‡ Institute of Genetics, Vetsuisse Faculty, University of Bern, 3001 Bern, Switzerland; §§ Mason Eye Institute, University of Missouri School of Medicine Columbia, Missouri

**Keywords:** progressive retinal atrophy, PRA, complex variant, retinal degeneration, whole genome sequencing, dog, palmitoyl protein thioesterase

## Abstract

Rod and cone photoreceptors are specialized retinal neurons that have a fundamental role in visual perception, capturing light and transducing it into a neuronal signal. Aberrant functioning of rod and/or cone photoreceptors can ultimately lead to progressive degeneration and eventually blindness. In man, many rod and rod-cone degenerative diseases are classified as forms of retinitis pigmentosa (RP). Dogs also have a comparable disease grouping termed progressive retinal atrophy (PRA). These diseases are generally due to single gene defects and follow Mendelian inheritance.We collected 51 DNA samples from Miniature Schnauzers affected by PRA (average age of diagnosis ∼3.9 ±1 years), as well as from 56 clinically normal controls of the same breed (average age ∼6.6 ±2.8 years). Pedigree analysis suggested monogenic autosomal recessive inheritance of PRA. GWAS and homozygosity mapping defined a critical interval in the first 4,796,806 bp of CFA15. Whole genome sequencing of two affected cases, a carrier and a control identified two candidate variants within the critical interval. One was an intronic SNV in *HIVEP3*, and the other was a complex structural variant consisting of the duplication of exon 5 of the *PPT1* gene along with a conversion and insertion (named *PPT1_dci_*). *PPT1_dci_* was confirmed homozygous in a cohort of 22 cases, and 12 more cases were homozygous for the CFA15 haplotype. Additionally, the variant was found homozygous in 6 non-affected dogs of age higher than the average age of onset. The *HIVEP3* variant was found heterozygous (n = 4) and homozygous wild-type (n = 1) in cases either homozygous for *PPT1_dci_* or for the mapped CFA15 haplotype. We detected the wildtype and three aberrant *PPT1* transcripts in isolated white blood cell mRNA extracted from a PRA case homozygous for *PPT1_dci_*, and the aberrant transcripts involved inclusion of the duplicated exon 5 and novel exons following the activation of cryptic splice sites. No neurological signs were detected among the dogs homozygous for the *PPT1_dci_* variant. Therefore, we propose *PPT1_dci_* as causative for a non-syndromic form of PRA (PRA*_PPT1_*) that shows incomplete penetrance in Miniature Schnauzers, potentially related to the presence of the wild-type transcript. To our knowledge, this is the first case of isolated retinal degeneration associated with a *PPT1* variant.

Rod and cone photoreceptors are specialized retinal neurons that have a fundamental role in visual perception, capturing light and transducing it into a neuronal signal. These light-sensitive cells are located adjacent to the retinal pigment epithelium (RPE), a cell layer that is vital for the maintenance and survival of photoreceptors. Aberrant functioning or altered development of rod and/or cone photoreceptors (and of their synaptic connections to second order neurons) or of the RPE often leads to dysfunction, progressive retinal degeneration and eventual blindness. Sequence variants in more than 300 genes and loci are associated to retinal degeneration in *Homo sapiens* (RetNet: https://sph.uth.edu/RETNET/ 2018), and an increasing number of comparable diseases have been described in non-human mammals. The latter can serve as valuable models for retinal disease gene discovery as they provide information regarding the precise molecular players and pinpoint the specific molecular mechanisms related to the severity and onset of the disease (Baehr and Frederick 2009, Miyadera *et al.* 2012). Large animal models of inherited retinal diseases have also proven useful in guiding the development of therapies that can then be transitioned to a clinical setting (Petit *et al.* 2016, Aguirre 2017).

In humans, many rod and rod-cone degenerative diseases are classified under the term retinitis pigmentosa (RP), a grouping that includes known and suspected diseases inherited as autosomal dominant, recessive or X-linked, as well as those of unknown mode of inheritance, which are referred to as simplex. Dogs suffer from a comparable group of diseases termed progressive retinal atrophy (PRA), and these diseases generally follow Mendelian inheritance, usually in an autosomal recessive manner - see for review: (Miyadera *et al.* 2012, Downs *et al.* 2013, Winkler *et al.* 2013, Downs *et al.* 2014; Svensson *et al.* 2016; Chew *et al.* 2017). Less frequent are those inherited as autosomal dominant (Kijas *et al.* 2002; Kijas 2003) or X-linked (Zhang *et al.* 2002; Kropatsch *et al.* 2016).

Photoreceptor dysplasia in Miniature Schnauzers, a more specific phenotype within the PRA group, was initially reported as an early-onset autosomal recessive disease characterized by abnormal development of rods and cones followed by rapid progression of degeneration. Breeding studies supported autosomal recessive inheritance with a high degree of statistical significance (Parshall *et al.* 1991). Candidate gene studies excluded the more obvious photoreceptor-specific/enriched candidates (Zhang *et al.* 1999) including a phosducin sequence variant (Zhang *et al.* 1998) that subsequently was found to not be disease-associated (Aguirre, unpublished information).

After identifying mutations in RPGR as responsible for X-linked PRA, we examined the breed origins of the XLPRA2 microdeletion found in mongrel-derived dogs in our research colony. As the research colony was intentionally outbred to increase polymorphisms for linkage mapping of disease (Acland *et al.* 1998; Aguirre and Acland 2006) we analyzed for the XLPRA2 microdeletion in a subset of dogs from each of the breeds that contributed to the colony population at the time; among these were included the extant population of photoreceptor dysplasia affected dogs together with archival DNA samples. This analysis confirmed the presence of the RPGR:c. 1084–1085delGA variant in hemizygous state in all affected male dogs, and in heterozygous state in all obligate female carriers (Zhang *et al.* 2002). A review of the phenotypes of dogs used for the breeding studies in the original publication (Parshall *et al.* 1991) indicated that XLPRA2 carrier females were clinically affected, but had a milder disease phenotype than hemizygous males, hence the misinterpretation of the breeding studies that indicated autosomal recessive inheritance. The disease, indeed, is X-linked semi-dominant, a feature also found in some X-linked RP human families with RPGR variants (Rozet *et al.* 2002).

The X-linked retinal degeneration in Miniature Schnauzers, termed Type A PRA, is associated with a c.1084–1085delGA in RPGR (Zhang *et al.* 1999). A commercial test for this variant was offered (OptiGen, LLC; http://www.optigen.com/opt9_test_a_pra.html). It soon became clear that Type A PRA was an extremely rare disorder in the breed; in the 17 years the test has been available, not a single case of the disease was found out of 375 samples tested. This prompted the company to provide free testing for Miniature Schnauzers, as well as other dog breeds, diagnosed with retinal degeneration. DNA from those Miniature Schnauzers provided the samples used in the current study aimed at the identification of additional genetic variants causing PRA in the breed.

## Materials and Methods

### Sample collection and phenotype assessment

Blood samples were obtained from 107 Miniature Schnauzers, 51 with a retinal degeneration compatible with a diagnosis of PRA, and 56 non-affected controls; all dogs were tested and found negative for Type A PRA. The average age of diagnosis for the cases was ∼3.9 ±1 years, the average age of examination for controls was ∼6.6 ±2.8 years. (Table 1). Samples were collected using standard clinical venipuncture techniques with full consent of the owners. The majority of these dogs resided in the United States, but additional dogs from Europe were also evaluated. In addition to the dogs clinically ascertained by one of the authors (GDA), the clinical evaluations of all the cases and controls were made by board certified veterinary ophthalmologists in North America (ACVO diplomates) and in Europe (ECVO diplomates), or accredited eye specialists. All examinations were done after pharmacologic mydriasis by indirect ophthalmoscopy and biomicroscopy as stipulated by the ACVO and ECVO guidelines for screening eye examinations. For a small subset of dogs fundus photographs were also available. Included with the blood samples were the eye examination records, clinical findings as well as, where possible, pedigree information. The clinical information provided with all samples was reviewed by an ACVO board certified veterinary ophthalmologist (GDA) prior to inclusion in the study.

**Table 1:** Summary table of the dogs involved in the project, and how many were genotyped with each SNP chip platform. Average age of diagnosis, and average age of the controls are also reported

Affected Miniature Schnauzers		51*
	Genotyped	***32***
	Homozygous CFA15 Haplotype	22
	Not Homozygous for CFA15 Haplotype	10
	Homozygous CFA15 Haplo in Illumina data	17
	Homozygous CFA15 Haplo in Affymetrix data	12
	In both	7
	No SNP chip genotype	***19***
		

Non-affected Miniature Schnauzers		56**
	Genotyped	***37***
	Heterozygous CFA15 haplotype	8
	No CFA15 haplotype	29
	No SNP chip genotype	***19***
	Homozygous *PPT1_dci_*	6

* Average age of diagnosis: ∼3.9 ±1.0 years.

** Average age of the controls: ∼6.6 ±2.8 years.

### SNP genotyping

DNA was extracted with the Illustra DNA extraction kit BACC2 (GE Healthcare) following manufacturer’s instructions. The first round of genotyping was carried out using the 48,341 marker Affymetrix canine SNP array. We genotyped 53 dogs, 24 cases and 29 controls. After a preliminary GWAS run, we then genotyped 27 dogs on the Illumina CanineHD Beadchip that included 173,661 evenly distributed SNPs. Of the 27 dogs genotyped on the second round, 11 (9 cases and 2 controls) had already been genotyped in the Affymetrix SNP chip. The remaining 16 dogs consisted of 8 additional cases and 8 additional controls (therefore 32 cases and 37 controls in total, Table 1). All SNP genotyping was carried out using standard protocols as recommended by the manufacturer.

### Genome-wide association

To carry out the GWAS, we merged the Illumina and Affymetrix data through the “–merge” and “–extract” commands with PLINK 1.9 (Chang *et al.* 2015). Then, we extracted SNPs with a genotyping rate >90% (“–geno 0.1”). We used BEAGLE3 (Browning and Browning 2007) to impute the missing SNPs, removed duplicate animals with PLINK (“–exclude”), and ultimately created the final dataset of 46,063 markers. This dataset was used for GWAS using the GenABEL package and the R Studio integrated development environment (Aulchenko *et al.* 2007). As a preliminary step for each analysis, we used standard quality control settings to remove markers and individuals with call rates <90% from the analysis. We also removed markers with minor allele frequency (MAF) <5%, and markers strongly deviating from Hardy-Weinberg equilibrium. Analysis of the pedigrees and breeding of the dogs in our cohort suggested that that the population is slightly stratified. This analysis was followed by mixed model association study as designed in the GenABEL package. The Manhattan plot was analyzed searching for suggestive or associated peaks.

### Homozygosity mapping and phasing

The GWAS analysis was followed by a homozygosity mapping approach in order to identify extended intervals of homozygosity with shared alleles as well as fine-map the region containing the responsible gene/genetic variant. Individuals and SNPs were selected using the commands “–keep” and “–extract”, while final files were generated through the “–merge” command. Homozygosity analysis was performed on all cases using the commands “–dog”, “–homozyg” and “–homozyg-group”, on the merged dataset. We used BEAGLE3 (Browning and Browning 2007) to phase the candidate chromosome and to look at the haplotypes of cases and controls. As the GWAS analysis pointed to a suggestive association peak in CFA15, we focused on this chromosome. The CFA15 file for BEAGLE was prepared with PLINK 1.9 using the “–beagle” and “–chr 15” commands. The haplotypes were visualized in Microsoft Excel.

### Remapping

Since the SNP chip data were based on the CanFam2.0 reference, and the mapping of the targeted sequencing and whole genome sequencing data were carried out against the CanFam3.1 reference, we used the NCBI “remap” function (https://www.ncbi.nlm.nih.gov/genome/tools/remap/) to convert the dataset based on CanFam2.0.

### Targeted sequencing

For targeted sequencing of the candidate region, we selected 6 PRA affected dogs (ages ranging from 2 to 4 years old), and 4 unaffected dogs (ages ranging from 3 years old to 11). For all 10 dogs, custom sequencing libraries were designed through Agilent SureDesign website (https://earray.chem.agilent.com/suredesign/), and biotinylated probes were used to capture DNA fragments in the candidate region with above 60% coverage. The custom sequencing paired-end libraries were prepared using Agilent SureSelectXT2 Target Enrichment System, and then sequenced by Illumina HiSeq2000 with a 300 bp insert size. The initial quality of the raw sequencing was checked by FastQC program (Brown *et al.* 2017). To obtain high quality variant calls, we followed the best practices guides for the most recent Genome Analysis Tool Kit (GATK) (McKenna *et al.* 2010). Specifically, raw reads were mapped to the dog reference genome CanFam3.1 using Burrows-Wheeler Aligner (BWA) v0.7.10 (Li and Durbin 2009), n = 8 threads. Duplicated reads were removed by Picard tools (https://broadinstitute.github.io/picard/). The unique aligned reads went through a local realignment to correct mismatched bases surrounding INDELs, and the base quality scores were recalibrated based on an empirically accurate per-base error model. BAM files containing the cleaned reads were analyzed by GATK HaplotypeCaller in which variant calling was strictly forced into the candidate region. Low quality variants were filtered based on a set of hard filters: (1) variant quality scores normalized by coverage (QD) <10; (2) overall mapping quality of reads (MQ) <40 and (3) strand bias (FS) >60.0. Variant calls with Mendel errors were removed by PLINK (Purcell *et al.* 2007). The above steps yielded 2,829 variants for downstream analysis. Out of these 10 dogs, 4 were also genotyped by Illumina arrays, we compared genotypes overlapping between two platforms. We found that percentages of consistent genotypes were > 96% across 4 samples, indicating a high calling accuracy of targeted sequencing analysis.

### Whole genome sequencing

Four Illumina TruSeq PCR free DNA libraries with a 350 bp insert size were prepared. The sequenced dogs were selected as two cases (homozygous for the CFA15 haplotype), one obligate carrier (parent) and a grand-parent. The cases were among those selected for the targeted sequencing. HiSeq2500 paired-end reads (2x 100 bp) were collected; the fastq files were created using Casava 1.8. A total of 1,010,526,513 100 bp paired-end reads were collected for the four dogs (∼250 million per dog corresponding to an average 15x coverage of the genome). The paired-end reads were then mapped to the dog reference genome CanFam3.1, and the reads aligned using Burrows-Wheeler Aligner (BWA) version 0.5.9-r16 (Li and Durbin 2009) with default settings. The SAM file generated by BWA was then converted to BAM and the reads sorted by chromosome using samtools (Li *et al.* 2009). The PCR duplicates were marked using Picard tools (http://sourceforge.net/projects/picard/).

### Variant discovery

The GATK version 2.4.9 program (McKenna *et al.* 2010) was was used for variant calling of aligned data, using the unified genotyper module of GATK. The variant data for each sample was obtained in variant call format (vcf, version 4.0), as were raw calls for all samples and sites flagged using the standard variant filtration module of GATK. Variant filtration was carried out, following the best practice documentation of GATK version 4. SnpEff software (Cingolani *et al.* 2012) together with the CanFam3.1 assembly was used to predict the functional effects of the variants detected. In addition, the Delly2 program (Rausch *et al.* 2012) was used to detect structural variants in the four BAM files, and to validate for suspected variants in a cohort of controls. The commands for deletions, insertions, inversion and duplications were all executed separately. The analysis focused on the candidate region. In order to avoid missing large inserts, deletions and false positives, all the variants detected were also compared within the candidate region using 192 control genomes (see File_S1, European Nucleotide Archive); note that in File_S1 Miniature Schnauzers are identified as ’Zwergschnauzer’.

### PCR and Sanger sequencing

The *PPT1* conversion (g.2,874,661_2,875,048con2,877,563-2,877,607inv, see below) was verified in all the available cases and carrier dogs by re-sequencing of targeted PCR products using Sanger sequencing. PCR primers were designed using PRIMER3 (Untergasser *et al.* 2012) after masking repetitive regions in the target region with RepeatMasker (http://www.repeatmasker.org/) and provided by Integrated DNA Technologies. A standard set of primers was designed for the *HIVEP3* intronic variant (g.1,432,293G > A) in order to genotype it. Additional steps were required in order to characterize the whole *PPT1* variant (*PPT1_dci_*, see below). Initially, because of the difficult nature of the region, three long PCR primers were designed in order to amplify the borders of the duplication (SequalPrep Long PCR, Thermo Fisher). The primers were two pairs. Each pair had the same common forward primer, and a different reverse primer. This resulted in a total of two distinct products (LF and LR1; LF and LR2, see File_S2): a longer 4368 fragment and a shorter 3641 fragment. In order to examine the *PPT1* exon 5 duplication variant, we first designed a PCR with primers targeted at the boundaries of the duplicated region (see the B1F, B1R, B2F, and B2R primers in File_S2). A specific primer set was designed to sequence the deleted interval (GF and GR). A complete characterization of the variant required Sanger sequencing of the long PCR products with B1R, B2F, GF, GR, LF, LR1 and LR2.

As a final step, in order to obtain an easily reproducible marker for detection of the variant, we carefully analyzed the BAM file and identified two consecutive variants falling within the duplicated interval: g.2,872,023delAG and g.2,872,103G > A (File_S5), and used them to genotype the remaining cases. To further validate these markers, we confirmed the presence (either in homozygosity or heterozygosity) of the conversion (g.2,874,661_2,875,048con2,877,563-2,877,607inv) with the GF and GR primers (File_S2), targeted on the deleted interval. Such primer pair produces a 538 bp fragment.

PCR was carried out with AmpliTaqGold360Mastermix (Life Technologies), and the products were sequenced using the PCR primers on an ABI 3730 capillary sequencer (Life Technologies) after treatment with exonuclease I (N.E.B.) and rAPid alkaline phosphatase (Roche). PCR products were run on 1% agarose gel with 0.5 μg/ml ethidium bromide. Sequence data were analyzed using Sequencher 5.1 (GeneCodes).

### Transcript characterization

Because of the almost ubiquitous expression of *PPT1*, RNA was extracted from the blood of a PPT1dci –homozygous case using the GeneJET RNA Purification Kit (Thermo Fisher). Reverse transcription was carried out with Superscript III as described by the manufacturer (Thermo Fisher), and PCR was carried out using AmpliTaqGold360Mastermix as described above. The primers were designed in order to span the exon 4 to exon 6 interval (**File_S2**). The PCR products were sequenced as above. The products were ligated to TOPO-TA cloning plasmids pCRII (Thermo Fisher), as described by the manufacturer, and the resulting vector was used to transform competent cells. The colonies grown overnight were collected with a pipette tip, and lysed in 20 µl of dH_2_O. Such solution was used as a template for sequencing with the same primers used for the cDNA exonic amplification.

The *HIVEP3* mRNA was annotated using SPLIGN (https://www.ncbi.nlm.nih.gov/sutils/splign/splign.cgi) with the human transcript NM_024503.4 as template.

### TPM calculation

In order to have additional information concerning the transcript expression for *HIVEP3* (ENSCAFT00000004055) and *PPT1* (ENSCAFT00000038806) in retinal tissue, we decided to search in three different canine retinal RNAseq datasets from outcrossed dogs that are not Miniature Schnauzers were used. The dataset consisted of 11, 7 and 9 dogs, used in different projects carried out in our group. Using the RNA-seq analysis software Kallisto (Bray *et al.* 2016), we calculated the average tpm (transcript per million) for the *HIVEP3* and *PPT1* canine transcripts within all three datasets.

### Data availability

File_S1: List and accession numbers of the 196 genomes of the study available in the European Nucleotide Archive. File_S2 – Primers used in the study. File_S3 – Orientation of the 45 bp interval copy. File_S4 – Targeted sequencing results for the region. File_S5 – Detail of the markers situated within the repeated region. File_S6 – The four transcripts detected by cloning and sequencing. File_S7 – Alignment of the wild type and mutant predicted protein. SNPchip and Targeted sequencing data are available in the Targeted_Sequencing_SNPchip.Murgiano file. Supplemental material available at Figshare: https://doi.org/10.25387/g3.6830633 and https://doi.org/10.25387/g3.6836858.

## Results

### Phenotypic characterization of a new PRA form in Miniature Schnauzers

Clinical examination of Miniature Schnauzers, and the archiving of blood samples for molecular studies continued after publication of the disease characterization and genetic analysis of photoreceptor dysplasia (Parshall *et al.* 1991). Once the mutation responsible for XLPRA2, *RPGR*:c.1084–1085delGA, was identified (Zhang *et al.* 2002), samples from all *RPGR* normal Miniature Schnauzer dogs diagnosed with PRA were archived. This resulted in 51 affected dogs with a presumably novel form of PRA. Based on the limited number of cases examined by one of the authors (GDA), PRA-affected dogs were clinically indistinguishable from the earlier described photoreceptor dysplasia cases (Parshall *et al.* 1991). Clinical examinations using biomicroscopy and indirect ophthalmoscopy showed that affected dogs were normal when examined at 10 months of age or earlier. Subsequently, fundus changes indicative of PRA developed. By 3 years of age, affected dogs showed advanced retinal disease. They had slow and incomplete pupillary light reflexes, and showed poor vision in familiar surroundings or preferred to be in a crate and not moving around. Comments were made on the clinical records of 3-year-old dogs examined by one of the authors (GDA) noted ’Fundus typical of photoreceptor dysplasia for age’.

**Figure 1:**
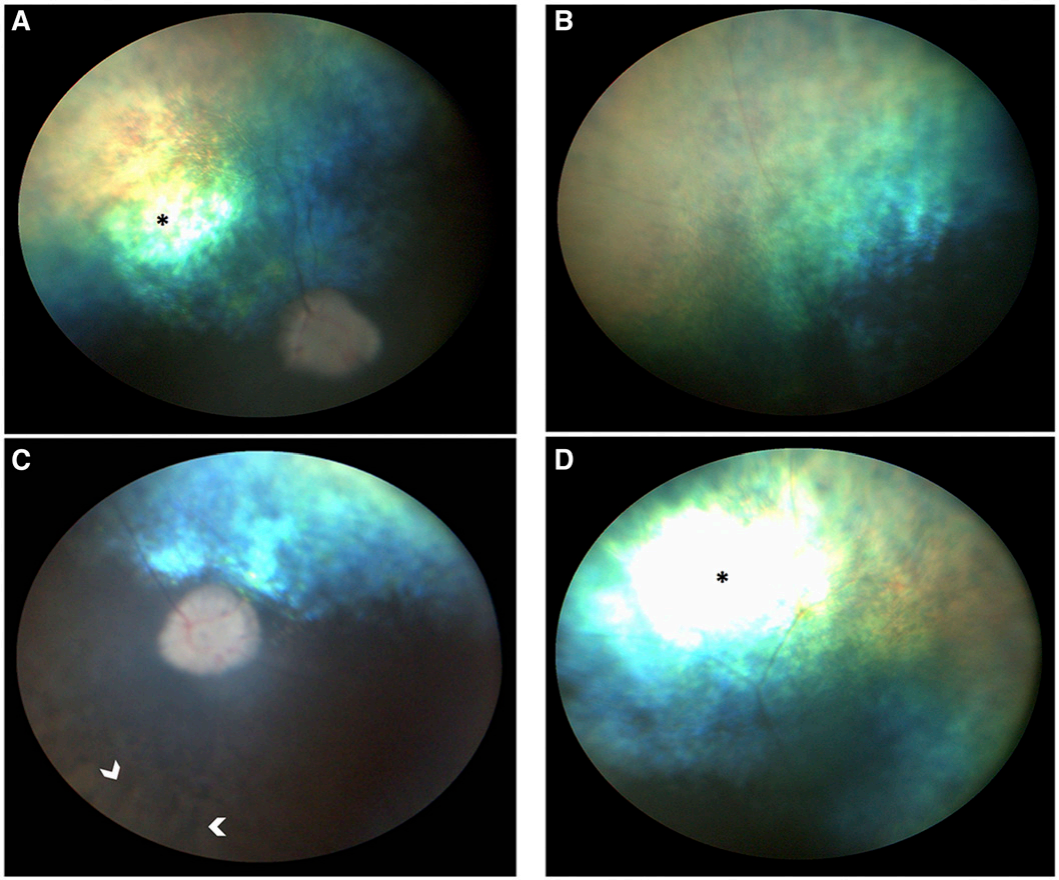
Fundus photographs of the right (A, B) and left (C, D) eyes of a 4 year old female Miniature Schnauzer homozygous for the *PPT1* complex structural variant. Both eyes show marked attenuation and loss of the major and minor retinal arterioles and venules, and the optic disc is pale and undergoing atrophic changes. With the proper angle of illumination, there is pronounced increased reflectivity from the tapetum lucidum (* in A and D), an indication of severe retinal thinning. The non-tapetal region shows patches of retinal pigment epithelial atrophy which appear paler, and these are situated adjacent to hyperpigmented and darker areas (C arrows). Photographs provided by Dr. Julien Charron, Clinique vétérinaire Saint Roch, La Rochelle, France

Fundus abnormalities at ∼3 years of age were characteristic of mid-stage disease (Parshall *et al.* 1991), consisted of marked attenuation or loss of retinal vessels, diffuse hyperreflectivity and ridging of the tapetal region (an indication of retinal thinning) and RPE loss and pigment migration in the non-tapetal regions of the eye (Figure 1). Cataracts were not present at this time. Progression to end-stage retinal atrophy and blindness occurred with age, although we could not specify the age when these changes occurred as most of the samples and clinical records were submitted from dogs prior to this advanced disease stage.

Most samples were received for molecular studies after a clinical diagnosis of PRA was made; the average age of the affected dogs was 3.9 ± 1 years of age. In fact, seven dogs were older, with clinical disease not diagnosed until 4.5-6.5 years of age. Unfortunately, based on the method of sample acquisition and the number of different examiners involved, it was not possible to obtain more specific details of the age of onset for PRA.

### Mapping of the PRA locus

**Figure 2:**
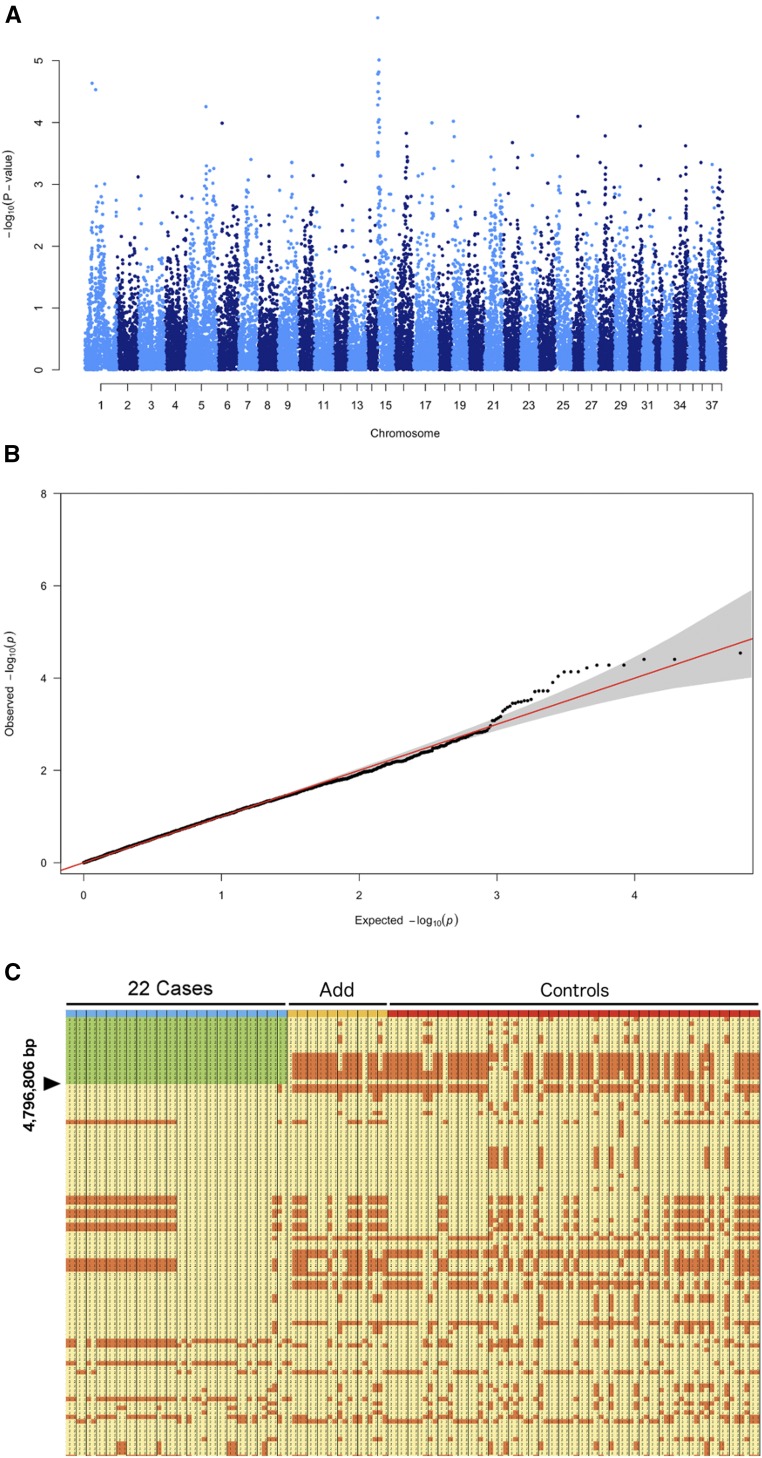
Mapping. (A) Results of the genome-wide association study (GWAS), obtained analyzing the imputed Affymetrix/Illumina merged data showing the negative log of the raw p-values calculated with the genotypic association test (max p-value 2.20e-06) (B) QQ-plot, showing the observed *vs.* expected log p-values. The moderate skewing of a marker toward the upper side suggests a weak association with the “affected” condition compared with what would be expected by mere chance. (C) Result of the homozygosity mapping using the 27,061 shared SNPs merged dataset: detail of the markers encompassing the first 14,490,600 Mb of CFA15. In green the candidate region shared exclusively among a sub-group of blue-marked 22 cases. Other cases (“Add”) are marked in yellow-orange, “Controls” in red. The region is shown in Microsoft Excel with the different alleles colored for ease of visualization. We opted to select a candidate region as the combination of the suggestive peak (log –pvalue 05) obtained by the GWAS and the homozygosity mapping.

**Figure 3:**
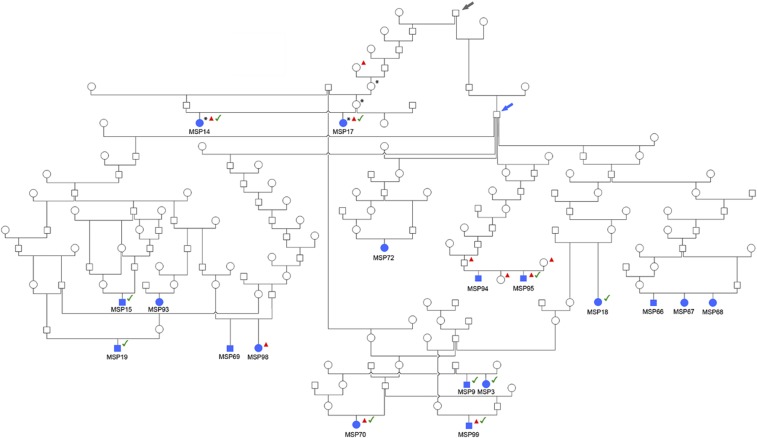
- Family tree of 18 Miniature Schnauzers cases affected with PRA in which there was sufficient family history to identify a putative common ancestor. Females are shown in circles, males in squares. All the animals indicated with the blue filled symbol are homozygous for the CFA15 haplotype. Dogs indicated with an asterisk (*) were used in WGS. Dogs indicated with a red triangle are cases and controls subjected to targeted sequencing. Dogs with the green tick have been confirmed homozygous for the *PPT1* variant through targeted or Sanger sequencing. The blue arrow shows a confirmed common ancestor for many cases, and putative carriers. The gray arrow shows another older, common ancestor in which the carrier status is definitive.

We carried out GWAS with the imputed 46,063 SNPs dataset consisting of 32 cases and 37 controls. After removing 18,679 non-informative markers and 2 controls on the basis of the thresholds (genotyping rate and Hardy-Weinberg equilibrium, described in the methods), 29,385 SNPs were used for genome-wide association mapping (GWAS). The results for this first GWAS are shown in Figure 2A. The calculated genomic inflation factor (lambda) was 0.98, which does not suggest a high stratification. The analysis did not show any significantly associated markers, but we detected a possible suggestive peak (max p-value 2.20x10^−6^) on CFA15. The QQ- plot suggested a difference between cases and controls (Figure 2B). Because of the imperfect segregation scenario (not all the cases shared the same haplotype) we opted to select a candidate region as the combination of the suggestive peak (log –pvalue 05) obtained by the GWAS and the homozygosity mapping. Based on GWAS, the best eight markers were located in the same single contiguous genomic region (3.43–7.93 Mb – first marker at position 3,435,908 (CanFam2.0)) on CFA15. The possibility of a monogenic autosomal recessive mode of inheritance was assessed by looking into the family history of the affected dogs. A sub-population of the affected animals could be identical by descent (IBD) for the causative variant and flanking chromosomal segments. We searched for extended regions of homozygosity with simultaneous allele sharing in the merged dataset. We found that 22 cases shared a genome region (greater than the established 1 Mb threshold) that fulfilled these criteria in CFA15, a chromosome we opted to focus on, in light of the suggestive peak detected in the GWAS. The remaining cases were not homozygous for the CFA15 haplotype (Figure 2C, Table 1). We remapped the positions of flanking markers of this candidate region. CFA15: 3,089,722 in CanFam2.0 was remapped to CFA15: 213,416 in CanFam3.1; while position CFA15: 7,806,737 was remapped to CFA15: 4,796,806 (Figure 2C). The mapped critical interval contains 102 annotated genes, 70 of which are protein coding, plus a number of uncharacterized loci. We carefully searched the data records of the dogs homozygous for the CFA15 haplotype and reconstructed their family tree. The reconstructed family information and most likely common ancestors are shown in Figure 3.

### Identification of candidate causative variants

Because of the extent of the candidate region and the large number of genes present, we opted to carry out targeted sequencing of the interval for variant detection. Therefore, we selected six cases homozygous for the disease-associated CFA15 haplotypes, and four controls (based on the family information, two of these controls were obligate carriers, see Figure 3). We detected 35 exonic variants of interest in the candidate interval. Of these, 15 were silent. We predicted the impact of the variants with the PolyPhen software (Adzhubei *et al.* 2010), and excluded all but two of the variants because they were predicted to be non-pathogenic. The two remaining, a g.339,815C > T variant in *LOC482436* (c.595C > T, p.Arg199Cys) and a g.3,209,909C > G variant in the *NT5C1A* gene (c.41C > G, p.Pro14Arg) were subsequently excluded as genotyping showed that they did not segregate with the disease.

For this reason, whole genome sequencing (WGS) was carried out, sequencing two affected animals, one obligate carrier and one control (Figure 3). The cases were included in the targeted sequencing of the candidate CFA15 region. The aim was to detect all the variants in the annotated genes and loci of the mapped 4.8 Mb interval on CFA15, and to potentially focus on other regions in affected dogs if detecting a plausible causative variant in the CAF15 critical interval failed. Sequencing, carried out in collaboration with the Dog Biomedical Variant Database Consortium (DBVDC), allowed us to filter and compare *any* called variant with the ones present in the database, and compare its frequency within the population of sequenced control dogs. Following whole genome sequencing and mapping, we compared and filtered SNVs as well as small indel variants detected in the cases with the variants in the controls and 192 publicly available dog genomes sequenced by DBVDC (File_S1).

**Figure 4:**
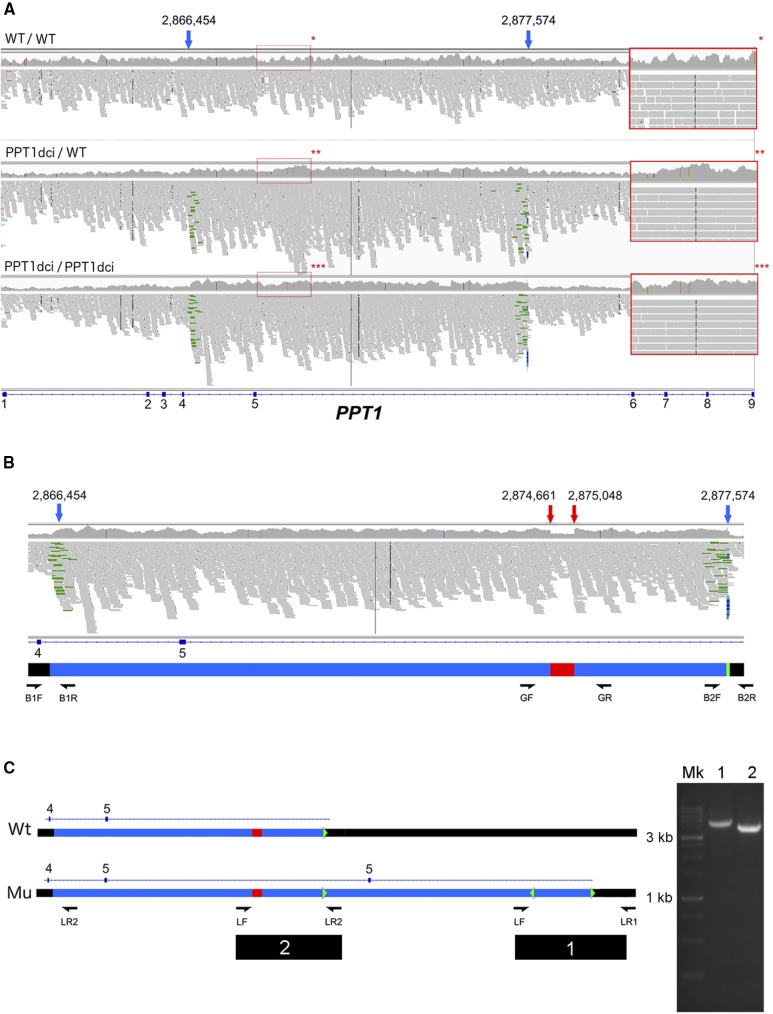
Characterization of the 11Mb-spanning variant. Figure is based on screenshots of the interval visualized with IGV. (A) Duplication of the 5^th^ *PPT1* exon visualized with IGV. The 11,120 bp duplication can be observed. From top to bottom, a case, a carrier and a control are shown. Note the inverted pairs, marked in green by IGV, on the variant boundaries present in carrier and case. Compare the coverage of the area with the flanking regions. The blue arrows show the mutation boundaries. In the red squares, a region for each of control (*), carrier (**) and case (***) is shown. Note the heterozygous SNPs contained in the carrier interval. Additionally, compare the coverage of the carrier with the flanking regions, and note the greater coverage of the case in comparison to carrier and control. (B) Critical regions of the variant. The blue arrows show the mutation boundaries (g.2866454_2877574dup), the red arrows the conversion (g.2874661_2875048con2,877,563-2,877,607inv). In detail, the green interval represents the 45 bp interval (2,877,563-2,877,607) duplicated, inverted and inserted in position g.2874661_2875048 in lieu of the bases normally present in such interval. Details for the sequencing primers are shown in File_S2. (C) Representation of the variant – top, the wild type (Wt), bottom the duplication (Mu). Observe the gap region (in red in the reference) missing in the first copy, substituted by an inserted and inversed copy of the 45 bp interval (2,877,563-2,877,607), shown as a green arrowhead. Primer placement for the long-range PCR is shown: on the right, the long PCR products obtained (1 and 2 are shown run on a 1% agarose gel, along with the ladder (Mk). See also File_S2.

Subsequently, through filtering processes described above, we identified a non-intergenic variant in our dataset that was homozygous in the affected dogs. This was a single intronic variant in the 3^rd^ intron of *HIVEP3* (CFA15, g.1,432,293G > A). *HIVEP3* is not, to our knowledge, associated with retinal diseases (Hicar *et al.* 2001), and has low retinal expression (Unpublished information; see below). Notably, the variants previously detected with targeted sequencing were filtered out in the WGS dataset. As we did not consider the intronic *HIVEP3* variant to be likely causative, we used the Delly2 package in search for structural variants in the sequenced dogs, as such variants would not have been detected by our initial analysis pipeline. In the cases, a total of 8 deletions, 4 inversions, 9 insertions and 8 duplications were found in the critical 4.8 Mb region. Following filtering, we observed that only a single duplication was present in the homozygous state in cases, but not in controls. This variant comprised a duplication of exon 5 of *PPT1*, in position 2,866,454-2,877,574, CanFam3.1 (Figure 4A). Further analysis revealed the variant to be more complex than a simple duplication; in fact, a deletion is present within the duplicated region in which the reference 2,874,661-2,875,048 interval is deleted (Figure 4B). Furthermore, the terminal part of the duplication had reads that suggested an additional inversion event (File_S3). We searched for the variant in a cohort of 192 additional control canine genomes (File_S1) and the variant was not present in any of them.

The tpm count analysis of mRNA highlighted the different expression levels of *HIVEP3* (ENSCAFT00000004055) and *PPT1* (ENSCAFT00000038806) in canine retina tissue in three independent RNA-seq datasets comprising 27 dogs. Transcripts from both genes were present in retina. For *HIVEP3*, we calculated a tpm average of 5.70 (9 samples), 2.15 (7 samples) and 7.59 (11 samples). For *PPT1*, we calculated a tpm average of 161.01 (9 samples), 155.78 (7 samples) and 119.01 (11 samples). Across all the samples, *PPT1* transcripts were present at 143 transcripts per million (tpm) and thus 25 times more abundant than *HIVEP3* transcripts (5.55 tpm).

### Characterization of a complex structural variant

We validated the boundaries of the variant as described in the Methods (Figure 4A, 4B). We also verified the position of the deletion as occurring in the first of the two duplicated segments (Figure 4C). Furthermore, we confirmed the presence of an interesting additional element, a boundary 45 bp segment mapping on the duplication border, exactly on the 2,877,563-2,877,607 interval. This fragment is duplicated, and then inserted in lieu of the deleted 2,874,661-2,875,048 interval (Figure 4B, green arrowhead, File_S3). Albeit significantly shorter than the reference, this last feature is not a simple deletion: it represents a conversion. In fact, an interval of nucleotides is replaced by a sequence from elsewhere in the genome. In this specific case, our converted fragment is inserted into the new position in a manner that is inverted in comparison to the original sequence. Ultimately, we defined the variant with respect to the CanFam 3.1 assembly as CFA15 g.[2,866,454_2,877,574dup; 2,874,661_2,875,048con2,877,563-2,877,607inv], and designated the mutant allele at this variant as *PPT1_dci_* for brevity.

We then re-evaluated the targeted sequencing data in light of the presence of the described *PPT1_dci_* rearrangement. We confirmed the presence of the *PPT1_dci_* allele in eight dogs used for targeted sequencing: in the six cases, the variant was homozygous, and heterozygous in the 2 obligate carriers (File_S4).

We selected two consecutive variants falling within the duplicated interval (Figure 4B) as markers. Using the GF and GR primers, we considered the dogs homozygous for *PPT1_dci_* only if their DNA produced the 538 bp fragment and also were homozygous for the g.2,872,023delAG and g.2,872,103G > A variants. We do not consider the presence of the 538 bp band on its own as indicative because it is visible whether the dog is homozygous or heterozygous.

### Genotyping of the variants within the population

We genotyped the *HIVEP3* and *PPT1_dci_* within the population of available Miniature Schnauzer samples. Concerning the *HIVEP3* intronic variant, it was not found homozygous in any control. This includes the controls reported above as homozygous for *PPT1_dci_*. Nonetheless, the variant was absent in four of the confirmed cases homozygous for *PPT1_dci_*. Of these, 3 cases were heterozygous and 1 was homozygous wild-type. The variant was also heterozygous in one additional PRA-affected case homozygous for the CFA15 haplotype in the homozygosity mapping. Based on these results, the *HIVEP3* intronic variant was excluded from causal association with the disease.

The disease-associated CFA15 haplotype was confirmed as homozygous in 22 PRA affected dogs (Table 1). We added 10 additional PRA-affected dogs from the general population to the 22 dogs described above (therefore, 32 dogs in total). We confirmed the presence of the *PPT1_dci_* in a total of 22 cases (Table 2). Three were confirmed exclusively through Targeted Sequencing. For the 10 remaining affected dogs with the CFA15 haplotype, we did not have enough DNA to experimentally confirm the *PPT1_dci_* structural rearrangement (Table 2).

**Table 2:** Summary table of genotyping results for *PPT1_dci_*. Affected and unaffected dogs homozygous and heterozygous for the haplotype and the *PPT1_dci_* variant are shown

*PPT1_dci_* Affected by PRA		22
	Haplotype CFA15 and Genotyped	10
	Genotyped	9
	Targeted Sequencing only	3
**PRA Affected, CFA15 haplotype but not genotyped for *PPT1***		10*
		
***PPT1_dci_* Unaffected by PRA**		**6**
		
Carriers confirmed in the sample population (total)		11
*-insufficient DNA for genotyping		

Of the 32 CFA15 homozygous/ *PPT1_dci_* cases described above, we identified 26 dogs affected with PRA who were >3 years of age at the time of the last ophthalmic examination (as reported in results; also 3 years corresponds to the lowest end of the absolute deviation from the average time at diagnosis recorded, see Table 1).

On the other hand, out of 56 controls, 6 dogs homozygous for *PPT1_dci_* were clinically normal (and >3 y.o.– the age range is infact between 3 and 10 years). The rest of the 50 non-affected controls used in this study were not homozygous for *PPT1_dci_*, or for the specific CFA15 disease-associated haplotype. We detected 17 heterozygous dogs and therefore potential carriers in the unaffected dog population.

The z-score for the association of the *PPT1_dci_* variant with the retinal disease (26 >3 years cases *vs.* 6 unaffected - all homozygous - for the variant or the haplotype) is 2.51, and the p-value of the association is 0.017.

Finally, 17 PRA cases in our dataset were not found to be not homozygous for the *PPT1_dci_* and/or the CFA15 haplotype, or for the *HIVEP3* intronic variant, suggesting the existence of at least one additional retinal degeneration locus in the breed.

### Characterization of the aberrant PPT1 transcripts

**Figure 5:**
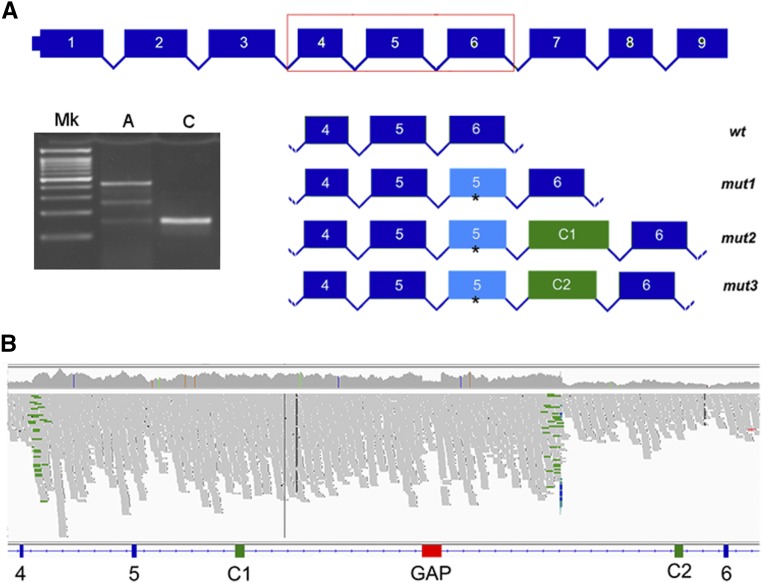
- Characterization of the transcripts. (A) PCR carried out with the 4F and 6R primers shown in File_S2. On the gel, is shown the ladder (Mk), RT-PCR of the blood cDNA extracted from one affected individual (A) is shown along with PCR obtained from the control (C). A schematic of (I) the wild-type transcript, *wt*; (II) a mutant transcript containing a retained exon 5 from the duplication (that is, containing thus two copies of the 5^th^ PPT1 exon), *mut 1*; (III) a third, longer transcript containing a second copy of the 5^th^ PPT1 exon plus an additional 166 bp fragment, *mut2*; (IV) a fourth, aberrant transcript containing a second copy of the 5^th^ PPT1 exon plus an additional 141 bp fragment, *mut3*. The asterisk shows the common premature stop codon in the additional exon 5. (B) Localization on the reference sequence (included an IGV screenshot of the interval) of the C1 and C2 novel exons, in position c.2,870,731-2,870,896 (C1) and 2,879,973-2,880,113 (C2), respectively.

Because retinal tissues from affected dogs in pre-degenerate disease stage were not available, we used freshly collected whole blood to characterize the expression of the widely expressed *PPT1* gene. After RNA extraction and cDNA synthesis from the blood of one case and one control, we designed a PCR targeting exon 4 and exon 6 of the *PPT1* transcript. The sequencing of the cloned amplicons allowed us to characterize the effect of the variant on the mutant *PPT1* transcripts. In the affected dog, we found the presence of four different types of transcript: (I) a wild-type transcript; (II) a mutant transcript containing a retained extra copy of exon 5 from the duplication (c.434_537dup); (III) a longer aberrant transcript containing a second copy of the 5^th^ *PPT1* exon, *plus* an additional 166 bp fragment; (IV) a longer aberrant transcript containing a second copy of the 5^th^ *PPT1* exon, *plus* an additional 141 bp fragment. Alignment of the fragments with the *PPT1* reference sequence revealed that the additional fragments in (III) and (IV) are the result of the inclusion of novel exons corresponding to positions chr15:2,870,731-2,870,896 and 2,879,973-2,880,113, respectively, due to the activation of novel cryptic splice sites (Figure 5, File S6). Due to the variability associated with the PCR conditions and the different lengths of these mutant transcripts, we cannot accurately quantify their presence in the original tissue. In the control sample, we detected only the wild-type transcript.

The resulting predicted protein sequences are reported in **File_S7**. The wild type protein is predicted to be 306 amino acids long. The duplicated exon 5 sequence (c.434_537dup) shared by all the three mutant transcripts generates a premature stop codon and is predicted to result in a frameshift and identical truncated translation products, p.(Arg180CysfsTer14). We did not experimentally assess whether this truncated protein is truly expressed.

## Discussion

### A coding PPT1 variant as a suitable candidate for PRA

In this paper, we provide evidence that homozygosity for the *PPT1_dci_* allele is strongly associated with PRA in the Miniature Schnauzer breed, and we refer to the disease as PRA*_PPT1_*. The use of GWAS and homozygosity mapping in combination with whole genome sequencing enabled us to identify a suitable candidate region. Access to sequenced genomes through the Dog Biomedical Variant Database Consortium (DBVDC) was imperative in the filtering and the proper selection of disease-associated variants, significantly reducing the number of variants falling within the candidate CFA15 region that required testing for the association. We identified a duplication encompassing roughly 11 kb on CFA15, which later revealed to be the more complex *PPT1_dci_*.

Of the 51 cases of PRA studied that were wildtype for the Type A PRA (*RPGR*) variant, the disease-associated CFA 15 haplotype was homozygous in 32 PRA affected dogs. We confirmed the presence of the *PPT1*_dci_ allele in 22 of them, but for the remaining 10, we did not have enough DNA to experimentally confirm the structural rearrangement. Six non-affected dogs were homozygous for the *PPT1_dci_* variant, and four of them were examined above the average age of diagnosis for the disease.

Another candidate variant that was initially considered is the *HIVEP3* CFA15 intronic variant, g.1,432,293G > A. The HIVEP proteins represent a group of polypeptides which are studied for their roles in the regulation of an assortment of genes, including those encoding collagen type IIA, αA-crystallin and β-interferon (Hicar *et al.* 2001). To our knowledge, *HIVEP3* is not associated with retinal disorders and retinal metabolism, nor does it have any known role in retinal structure or function. Additionally, by analyzing the tpm of the transcripts of both genes in three different datasets, we found that *PPT1* transcripts are ∼25 times more abundant compared to *HIVEP3*. The *HIVEP3* intronic variant has been found heterozygous (3 times) or wild type (1 time) in four cases homozygous for the *PPT1_dci_* variant. Furthermore, it was found heterozygous in a fifth unconfirmed case homozygous for the CFA15 haplotype.

*PPT1_dci_* is (I) the only homozygous variant occurring in all the PRA cases homozygous for the CFA15 haplotype; (II) involves a gene known to have a role in (syndromic) retinal degeneration in dog and other species; and (III) is shown to be highly expressed in retina compared to *HIVEP3*. Therefore, we propose the *PPT1_dci_* variant as causative for PRA in Miniature Schnauzer. Due to the presence of six clinically normal homozygous mutant dogs, we postulate incomplete penetrance of the phenotype for the variant. This hypothesis is consistent with the non-syndromic nature of the retinal disease.

The presence of the *HIVEP3* variant in heterozygosity in a homozygous interval is unexpected.-Nonetheless, the lack of perfect segregation between the two variants suggests that a recombination probably occurred in a previous generation. Cases with similar discrepancies (perhaps suggesting a different explanation such as additional gene conversion events, or even potential revertation mutations) are not unheard of and have previously been reported (Gerber *et al.* 2015). It is also possible that the lack of higher density SNP information prevented us from discerning what are in fact two different haplotypes in the acrocentric region of CFA15.

### Role of PPT1 in retinal degeneration

The *PPT1* gene encodes for palmitoyl-protein thioesterase 1 (PPT1), a small glycoprotein and lysosomal hydrolase that catalyzes the removal of palmitate from its thioester linkage to serine residues in S-acylated proteins. PPT1 has been localized to lysosomes in some cell types (Hellsten *et al.* 1996). However, Heinonen and colleagues suggested that PPT1 may not exclusively be a lysosomal hydrolase, and that the specific targeting of PPT1 into the neuritic shafts and nerve terminals indicates that PPT1 may be associated with the maintenance of synaptic function as well. They also suggested the presence of an extracellular substrate for PPT1 (Heinonen *et al.* 2000). In murine primary neurons and brain tissue, PPT1 is localized in synaptosomes and synaptic vesicles but not in lysosomes, and, as such, abnormal synaptic functioning could play a role in the CLN1 form of neuronal ceroid lipofuscinosis (Lehtovirta 2001). Thus, it appears that PPT1 may play a different role in different cell types.

Homozygosity in over 80 variants in human *PPT1* result in the CLN1 form of neuronal ceroid lipofuscinosis (NCL), a progressive early onset neurodegenerative disease (http://www.ucl.ac.uk/ncl/CLN1mutationtable.htm). NCL associated with *PPT1* variants have also been reported in dogs (Sanders *et al.* 2010; Kolicheski *et al.* 2017). NCLs are a group of inherited lysosomal storage disorders, characterized by progressive neurodegeneration, including progressive retinal degeneration, and by the accumulation of autofluorescent lysosomal storage granules in the CNS and other tissues (Bennett and Hofmann 1999). In CLN1 disease, existence of a positive feedback loop has been proposed, where palmitoylation of PPT1 results in decreased activity and a subsequent elevation in the amount of palmitoylated proteins occurs. Such feedback is likely to initiate a vicious cycle that ultimately enhances NCL progression (Segal-Salto *et al.* 2016).

In humans, NCL-associated *PPT1* genetic variants vary in nature and position. Of 61 genetic variants analyzed, these ranged from missense (n = 27), nonsense (11), splice-site (10), insertions (4), deletions (6), insertion-deletion (1), one that could be either missense or splice-site affecting, and one variant affecting the initiator methionine (Kousi *et al.* 2011). Disease causing variants have been identified in each of the nine exons in humans. There is a correlation between the nature of the genetic variant and the sub-type of CLN1. In fact, combinations of nonsense or frameshift alleles result in an infantile onset phenotype with disease onset at less than 2 years and rapid progression (Das *et al.* 1998; Das *et al.* 2001). Overall, *PPT1* variants causing the more severe infantile and late infantile NCLs are located closer to the catalytic triad (Ser 115, Asp 233 and His 289) (Bellizzi *et al.* 2000; Das *et al.* 2001), and cause the most drastic reduction of the enzyme activity. In contrast, variants associated with later disease onset NCL are located further from the catalytic triad and predicted to cause less dramatic changes to secondary and tertiary structure of the protein (Das *et al.* 2001). Reports of milder phenotypes associated with missense variants suggest the use of alternative sorting pathways for PPT1, or the possibility that mutant PPT1 molecules show a higher degree of oligomerization, possibly as an attempt to regulate activation and/or transport of the enzyme (Lyly *et al.* 2007).

In mice, knockdown of *Ppt1* leads to spasticity and motor abnormalities, leading to death by 10 months of age (Gupta *et al.* 2001). Deletion of exon 4 replicates infantile NCL, and homozygous mutant mice show loss of vision from 8 weeks of age, seizures after 4 months and paralysis of hind limbs starting at 5 months (Jalanko *et al.* 2005). Mice with an inserted premature stop codon in exon 5 show hindlimb spasticity and granular osmiophilic deposits in brain and spleen, along with retinal degeneration (Bouchelion *et al.* 2014). Similarly, *PPT1* genetic variants in dog are also associated with neurologic abnormalities and blindness. In the Dachshund and Cane Corso breeds, a homozygous 1 bp insertion in exon 8 or a homozygous splicing site variant affecting exon 1, respectively, result in neurologic disease and early onset blindness (Sanders *et al.* 2010; Kolicheski *et al.* 2017).

Based on the association between *PPT1* genetic variants and NCL in patients and animal models, how can we explain the non-syndromic nature of the retinal disease in Miniature Schnauzer dogs with the complex *PPT1* exon 5 duplication variant? We hypothesize that this results from the high demand for rhodopsin (Rh) palmitoylation in the retina, and the presence of wild type mRNA that fulfills the needs for this enzyme in non-retinal tissues (Xiong and Bellen 2013). After all, heterozygous *PPT1* carriers, either human or animals, are normal in terms of neurologic function and vision (Sanders *et al.* 2010; Kousi *et al.* 2011; Kolicheski *et al.* 2017). The expression analysis on RNA isolated from peripheral white blood cells demonstrated that a proportion of *PPT1* wildtype transcripts, in addition to three aberrant transcripts, was present. This suggests that the variant, albeit spanning more than 11 kb, does not completely impede the expression of a wild type protein.

In the retina, Rh is subjected to a number of post-translational modifications (St Jules *et al.* 1990) of which cysteine S-palmitoylation is crucial for its biological function; depalmitoylation has been shown to inhibit Rh regeneration, and to increase its ability to activate transducin’s light-dependent GTPase activity (Morrison *et al.* 1991). Palmitoylated Rh can acquire raftophilicity upon G protein-stabilized dimerization, and thereby organize receptor-cluster rafts by recruiting raftophilic lipids, ultimately initiating the phototransduction cascade in rod photoreceptors (Seno and Hayashi 2017). There is evidence of cross-talk between the autophagic and endosomal/lysosomal pathways. In Drosophila, it has been shown that degradation of Rhodopsin 1 occurs via cross-talk between the autophagy and endosomal degradation pathways, and that autophagy acts as a compensatory pathway when the endosomal/lysosomal pathway is impaired (Midorikawa *et al.* 2010; Seno and Hayashi 2017). The study also indicated that Ppt1 is essential for photoreceptor viability in Drosophila, and Ppt1 mutants show light- dependent retinal degeneration secondary to intracellular accumulation of rhodopsins from Ppt1 disruption.

A similar finding of non-syndromic retinal disease, either isolated macular degeneration or widespread generalized retinal disease, has been reported in patients with the CLN7/*MSFD8* form of NCL. Different genetic variants in the gene were found and some were considered ’mild’ or ’moderate’ disease alleles. There were even patients who were affected with non-syndromic retinal disease when a null variant allele was present. In fact, as an example, in a previous study concerning predictive analyses of pathogenicity scores of variants on protein sequence, the authors found no correlation between the impact of the different variants and whether patients would develop non-syndromic retinal disease or NCL (Khan *et al.* 2017).

In regard to a possible late-onset neurologic disease in older dogs homozygous for *PPT1* variants, we were able to contact owners as well as referring veterinarians and follow up five older dogs homozygous for the *PPT1* complex variant and affected with PRA*_PPT1_*. These dogs were alive at the time, or had died from unrelated diseases (*e.g.*, heart failure). At ages ranging from 6-8 years, these dogs did not show any neurological disease. We do not possess data that is able to support a complete hypothesis regarding why *PPT1_dci_* has a retina-specific effect. However, we can speculate that the rate of outer segment (rhodopsin) turnover in retina could have a specific role, and the variant would affect the amount of the expression only on a level affecting the specific tissue with high requirements for palmitoylation, such as the retina (Aguirre and O’Brien 1986; Wetzel *et al.* 1989; St Jules *et al.* 1990; Katz *et al.* 1999); for this reason, the presence of the wild-type transcript would lead to a phenotype milder on a general level, explaining the non-syndromic nature of the disease.

Following a similar line of reasoning, we could suggest that the healthy dogs homozygous for the risk allele might produce a greater amount of wild-type transcript, or possibly a greater amount of such transcript is translated due to internal factors, such as the modulating influence of additional genetic variants *in cis* or *in trans* (Schubert *et al.* 1997; Raj *et al.* 2010; Cooper *et al.* 2013). The individual differences described above could explain the incomplete penetrance of the retinal disease.

In summary, our studies confirm a complex structural *PPT1* variant associated with a non-syndromic canine retinal degeneration (PRA*_PPT1_*). The characterization of the variant through whole genome and Sanger sequencing helped to identify a complex variant involving instances of duplication, deletion and re-insertion of a duplicated 45 bp fragment in a non-adjacent region. This genetic variant accounts for ∼63% of Miniature Schnauzers affected with retinal degeneration in which the Type A form of the disease has been excluded, which indicates that at least one additional retinal disease locus exists in the breed.
